# *Shilajit*: A Natural Phytocomplex with Potential Procognitive Activity

**DOI:** 10.1155/2012/674142

**Published:** 2012-02-23

**Authors:** Carlos Carrasco-Gallardo, Leonardo Guzmán, Ricardo B. Maccioni

**Affiliations:** Laboratory of Cellular and Molecular Neurosciences, International Center for Biomedicine (ICC) and University of Chile, Millennium Building, Las Encinas 3370, Ñuñoa, 780023 Santiago, Chile

## Abstract

*Shilajit* is a natural substance found mainly in the Himalayas, formed for centuries by the gradual decomposition of certain plants by the action of microorganisms. It is a potent and very safe dietary supplement, restoring the energetic balance and potentially able to prevent several diseases. Recent investigations point to an interesting medical application toward the control of cognitive disorders associated with aging, and cognitive stimulation. Thus, fulvic acid, the main active principle, blocks tau self-aggregation, opening an avenue toward the study of Alzheimer's therapy. In essence, this is a nutraceutical product of demonstrated benefits for human health. Considering the expected impact of *shilajit* usage in the medical field, especially in the neurological sciences, more investigations at the basic biological level as well as clinical trials are necessary, in order to understand how organic molecules of *shilajit* and particularly fulvic acid, one of the active principles, and oligoelements act at both the molecular and cellular levels and in the whole organism.

## 1. Introduction

*Shilajit* also known in the north of India as *salajit*, *shilajatu*, *mimie*, or *mummiyo* is a blackish-brown powder or an exudate from high mountain rocks, especially in the Himalayans mountains between India and Nepal, although it has been also found in Russia, Tibet, Afghanistan, and now in the north of Chile, named as *Andean Shilajit *[1]. *Shilajit *has been known and used for centuries by the *Ayurvedic* medicine, as a rejuvenator and as antiaging compound. There are two important characteristics of a *rasayana* compound in the ancient Indian *Ayurvedic* medicine: that is, to increase physical strength and to promote human health [2]. The health benefits of *shilajit* have been shown to differ from region to region, depending on the place from which it was extracted [3, 4].

## 2. Origins of *Shilajit*

Considering its unique composition as a phytocomplex, very rich in fulvic acid, researchers hypothesize that *Shilajit* is produced by the decomposition of plant material from species such as *Euphorbia royleana* and *Trifolium repens* [4, 5]. This decomposition seems to occur through centuries, and on this basis, *shilajit* is considered a millenary product of nature. However, further studies have identified that several other plant organisms may generate *shilajit*, such as molds as *Barbula, Fissidens, Minium, and Thuidium* and other species like *Asterella, Dumortiera, Marchantia, Pellia, Plagiochasma, *and* Stephenrencella-Anthoceros* [4].

## 3. Molecular Composition of *Shilajit*

**Figure 1:**
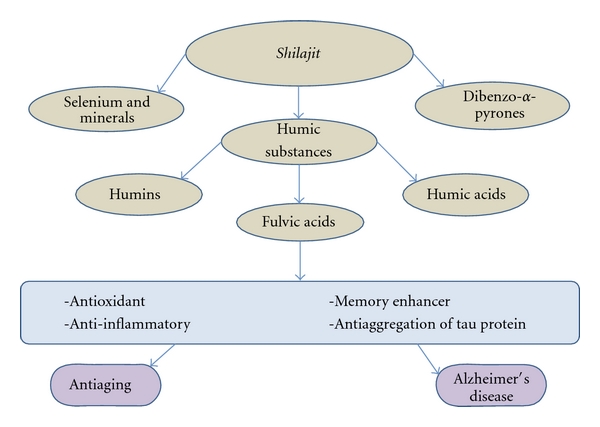
*Shilajit*, its main components, and potential uses based on properties of fulvic acid. This phytocomplex known as *shilajit* is mainly composed of humic substances. One of them, fulvic acid, is known by its properties such as antioxidant, anti-inflammatory, and memory enhancer. Novel investigations indicate that fulvic acid is an antiaggregation factor of tau protein *in vitro *[1], which projects fulvic acid as a potential anti-Alzheimer's disease molecule.

*Shilajit* is composed mainly of humic substances, including fulvic acid, that account for around 60% to 80% of the total nutraceutical compound plus some oligoelements including selenium of antiaging properties [6, 7] (Figure 1). The humic substances are the results of degradation of organic matter, mainly vegetal substances, which is the result of the action of many microorganisms. Components are divided operationally in humins, humic acid, and fulvic acids according to their solubility in water at different pH levels. Humins are not soluble in water under any pH condition. Humic acid is soluble in water under alkaline conditions and has a molecular weight of 5–10 kDa. Fulvic acid is soluble in water under different pH conditions, and because of its low molecular weight (around 2 kDa), it is well absorbed in the intestinal tract and eliminated within hours from the body [8, 9]. It is likely that the curative properties attributable to *shilajit* are provided by the significant levels of fulvic acids that *shilajit *contains, considering that fulvic acid is known by its strong antioxidant actions [9] and likely has systemic effects as complement activator [10]. Recent studies on the composition of Andean *Shilajit *in Chile have evidenced an ORAC index between 50 and 500 Trolox units/g of material, which is substantially higher than Noni and blueberries (Quinteros et al., unpublished data). In this context, *shilajit* seems to be a powerful antioxidant phytocomplex.

Other molecules present in *shilajit* preparations are eldagic acid, some fatty acids, resins, latex, gums, albumins, triterpenes, sterols, aromatic carboxylic acids, 3,4-benzocoumarins, amino acids, polyphenols, and phenolic lipids [3, 6, 11]. Certainly its molecular composition varies from region to region. Newer investigations based on high-performance size exclusion chromatography (HP-SEC) show that *shilajit *contains specific molecular species of polysaccharides and lignins [10]. As humic components, humins, humic acids, and fulvic acids are found in all *shilajit* preparations, being the last one, fulvic acids, the biologically active compound, along with dibenzo-*α*-pyrones, which acts as carrier of other substances [3]. 

## 4. Traditional Uses of *Shilajit*

*Shilajit *is an important, known component of the ayurvedic medicine given its characteristics as a *rasayana*. In this context, health benefits such as an increase in longevity, rejuvenating, and arresting aging roles have been attributed to it [3]. Traditionally, *shilajit *is consumed by people from Nepal and the North of India, and children usually take it with milk in their breakfast. The *Sherpas* claim to have *shilajit* as part of their diet; they constitute a population of strong men with very high levels of a healthy longevity. Our laboratory has found evidence on the high activity of the Andean form of *shilajit* in improving cognitive disorders and as a stimulant of cognitive activity in humans [1] (Table 1).

 Considering the actions of fulvic acid in preventing tau self-aggregation into pathological filaments, this compound appears to be of interest for prevention of Alzheimer's disease [1]. Other common traditional uses include its action in genitourinary disorders, jaundice, digestive disorders, enlarged spleen, epilepsy, nervous disorders, chronic bronchitis, and anemia [2]. *Shilajit* has been also useful for the treatment of kidney stones, edema, and hemorrhoids, as an internal antiseptic, and to reduce anorexia. Also, it has been claimed in India to be used as *yogavaha* [12, 13], that is, as synergistic enhancer of other drugs. Organic components of *shilajit* play also a role in transporting different mineral substances to their cellular targets.

## 5. Novel Investigations

Preclinical investigations about *shilajit* indicate its great potential uses in certain diseases, and various properties have been ascribed, including (1) antiulcerogenic properties [14]; (2) antioxidant properties [15, 16]; (3) cognitive and memory enhancer [1, 10, 17]; (4) antidiabetic properties [18]; (5) anxiolytic [12]; (6) antiallergic properties and immunomodulator [2, 19, 20]; (7) anti-inflammatory [21]; (8) analgesic [16]; antifungal properties [22]; (9) ability to interact positively with other drugs [23]; (10) protective properties in high altitudes [24]; (11) neuroprotective agent against cognitive disorders [1, and Farias et al. unpublished clinical trials]. Unfortunately *shilajit* lacks systematic documentation and well-established clinical trials on its antioxidative and immunomodulatory actions in humans, and it is expected that considering the reported benefits evidenced from trials will be obtained in the near future [25]. 

## 6. Patenting

A few patents already exist that protect the use of *shilajit* in India and Nepal, such as US Patent 5,405,613—vitamin/mineral composition [26]; US Patent application number 20030198695—Herbo-mineral composition [27]; US Patent number 6,440,436—Process for preparing purified *shilajit* composition from native *shilajit* [28]; US Patent number 6,558,712—Delivery system for pharmaceutical, nutritional and cosmetic ingredients [29]. Other recent patent about a phytocomplex with vitamins added is WO 2011/041920 [30].

## 7. Potential Risks

Studies indicate the *shilajit *consumption without preliminary purification may lead to risks of intoxication given the presence of mycotoxin, heavy metal ions, polymeric quinones (oxidant agents), and free radicals, among others [3]. Therefore, a purified, ready-for-use preparation for human consumption must be used. However, recent studies indicate that several ayurvedic products including *shilajit* and other Indian manufactured products commercialized by the Internet may contain detectable heavy metals levels as lead, mercury, and arsenic [31]. This study showed the presence of heavy metals and other minerals, including gems, is associated with the belief that when mixed with *shilajit* or other herbal preparations they generate a better response from the body in a synergic manner. This is what is known as *rasa-shastra* in ayurvedic medicine. *Rasashastra* experts claim that if this is prepared, administered, and consumed properly, it is safe and has therapeutic advantages [31]. It is worth considering that recent clinical reports indicate cases of lead poisoning in patients who have used ayurvedic products against weakening [32, 33].

## 8. Commentary and Discussion

*Shilajit* has a comfortable position as the *rasayana *because of its excellence, well known in the Eastern culture, and now being introduced with great interest in the occidental world. The vast majority of published papers on this theme are from India, leaving this sector of the planet as an expert in their field, since this is a product that is extracted, marketed, and investigated in these latitudes. However, this generates a segmentation of *shilajit*, relegating it only to what has always been assumed: a natural product that is part of natural alternative medicine and not as a result of medical and biotechnology innovation worldwide. This is evidenced quite clearly by reviewing the literature today, and note that the journals where studies on *shilajit *are published (jobs are plentiful) are mainly reviewed in the Eastern. Given this, it is necessary that *shilajit* break the cultural paradigm and enter into the rest of the world by the hand of rigorous research at the molecular and cellular levels, which could elucidate the interactions of the active ingredients of the different *shilajit* preparations with biomolecules. This will facilitate our understanding of their mechanisms of action.

## 9. Conclusion

*Shilajit* is a potent and very safe dietary supplement, potentially able to prevent several diseases, but its main medical application now appears to come from its actions in benefit of cognition and potentially as a dietary supplement to prevent Alzheimer's disease. In essence, this is a nutraceutical product. Considering the expected impact of *shilajit* applications in the medical field, especially in neurological sciences, more investigations at the basic biological level are necessary, and certainly well-developed clinical trials, in order to understand how its active principles act at molecular and cellular levels.

## Figures and Tables

**Table 1:** Morphometric study of primary cultured rat hippocampal cells exposed to *Shilajit* and the Brain Up-10 *formulae* that contain *Shilajit *plus complex B vitamins (Vit B6, B9, and B12).

	Control	Shilajit**	Brain Up-10*
Neuronal cells per field	367 ± 23	345 ± 42	396 ± 16.0
Percentage of cells with neuronal processes	18.0 ± 2.1	26.0 ± 3.2**	43.0 ± 3.1**
Fraction of axon-like processes	0.22	0.29	0.41
Processes length (*μ*m)	17.4 ± 7.2	26.0 ± 4.5**	39.6 ± 8.0**

Hippocampal cells were grown in Petri dishes in the presence of either 10 mg/mL *Shilajit* or the formulation of Brain Up-10 [30] plus vitamins of the B complex. In the control, cells were grown in culture medium without *Shilajit *or the formulation. Mean of 5 determinations (*n* = 5) (significance of differences with respect to control, **P < 0.001).
